# Bridging Mind and Heart: Inflammatory Signatures in Post-AMI PTSD Patients

**DOI:** 10.1192/j.eurpsy.2026.11873

**Published:** 2026-07-31

**Authors:** K. M. Panoutsopoulou, A. Grammeniati, N. S. Bastas, M. Gouva, A. Naka, E. Dragioti

**Affiliations:** 1 Department of Nursing, Scientific Laboratory of Psychology and Person-Centered Care; 2 Department of Medicine, Second Department of Cardiology, School of Health Sciences, University of Ioannina, Ioannina, Greece

## Abstract

**Introduction:**

Post-traumatic stress disorder (PTSD) develops in approximately 10–20% of patients following acute myocardial infarction (AMI). Emerging evidence suggests that PTSD may drive persistent inflammatory activation —marked by elevated C-reactive protein (CRP), interleukin-6 (IL-6), interleukin-1β (IL-1β), and matrix metalloproteinase-9 (MMP-9) potentially increasing the risk of recurrent ischemic events and worsen prognosis.

**Objectives:**

To synthesize recent evidence on associations between PTSD and inflammatory biomarkers among AMI survivors, and to examine whether inflammatory dysregulation may contribute to recurrent cardiac events or adverse outcomes.

**Methods:**

A systematic PubMed search (January 2015–September 2025) combined MeSH and free-text terms for PTSD, AMI, and inflammatory biomarkers. The search identified 39 unique records; after screening and full-text review, 12 studies met inclusion criteria. Eligible studies were human cohort or case–control designs assessing both PTSD and inflammatory or cardiovascular outcomes after AMI.

**Results:**

Across included studies, PTSD was consistently associated with elevated inflammatory biomarkers, particularly CRP, IL-6, and MMP-9. In one cohort of 271 AMI survivors, PTSD patients showed a 126% IL-6 increase after stress versus 63% in non-PTSD participants (p = 0.001). In a larger cohort (n = 3,049), PTSD was associated with higher odds of CRP >3 mg/L (OR = 2.27, 95% CI 1.32–3.93) and increased risk of recurrent infarction (HR = 1.42, 95% CI 1.33–1.50).

**Conclusions:**

PTSD following AMI appears associated with increased systemic inflammation and greater recurrence risk. Integrating psychocardiological care and early PTSD screening may improve outcomes. Further longitudinal studies should clarify mechanisms and test targeted interventions.

**Disclosure of Interest:**

None Declared

